# Benchmarking large language models against human experts in rehabilitation medicine: a multidimensional evaluation

**DOI:** 10.1186/s12984-026-01903-0

**Published:** 2026-03-02

**Authors:** Wenhui Cao, Mengjian Qu, Tao Zhu, Jing Liu, Ying Shen, Jihua Zou, Yi Li, Haiming Wang, Lisha Zhang, Huifang Liu, Qi Wu, Guijuan Zhou, Guanghua Sun, Helin Gong, Yaping Wan, Xiaofeng He, Jun Zhou

**Affiliations:** 1 Department of Rehabilitation, The First Affiliated Hospital, Hengyang Medical School, University of South China, Hengyang, 421001 Hunan China; 2 School of Computer Science, University of South China, Hengyang, 421001 Hunan China; 3 Department of Rehabilitation Sciences, The Hong Kong Polytechnic University, Kowloon, Hong Kong SAR China; 4 Faculty of Health and Social Sciences, The Hong Kong Polytechnic University, Kowloon, Hong Kong SAR China; 5 Department of Rehabilitation Medicine, The First Affiliated Hospital with Nanjing Medical University, Nanjing, 210029 Jiangsu China; 6 Department of Rehabilitation Medicine, Zhujiang Hospital, Southern Medical University, Guangzhou, 510280 Guangdong China; 7 Department of Rehabilitation Medicine, Rehabilitation Medicine Center, Rehabilitation Key Laboratory of Sichuan Province, West China Hospital, Sichuan University, Chengdu, 610041 Sichuan China; 8 Rehabilitation Medicine Department, The First Affiliated Hospital of Zhengzhou University, Zhengzhou, 450052 Henan China; 9 Department of Rehabilitation Medicine, Sichuan Provincial People’s Hospital, School of Medicine, , University of Electronic Science and Technology of China, Chengdu, 610072 Sichuan China; 10 SJTU Paris Elite Institute of Technology, Shanghai Jiao Tong University, Shanghai, 200240 China; 11 Department of Clinical Medicine, Suzhou Vocational Health College, Suzhou, 215000 Jiangsu China

**Keywords:** Large language models, Rehabilitation medicine, Clinical decision support, Human-AI collaboration, Benchmarking, Performance evaluation, Multidimensional evaluation framework, Rehabilitation plan

## Abstract

**Background:**

Rehabilitation medicine faces a significant challenge due to the rising demand for services coupled with a shortage of specialized professionals. Large Language Models (LLMs) show promise for enhancing clinical efficiency, but their evaluation has been largely limited to simulated scenarios, lacking direct performance comparisons with human experts in complex, real-world clinical tasks.

**Objective:**

To systematically benchmark five state-of-the-art LLMs against senior physiatrists in formulating comprehensive rehabilitation plans for authentic clinical cases, evaluating their utility as clinical decision support tools.

**Methods:**

We conducted a rigorous, blinded evaluation using 48 authentic cases across six subspecialties. Plans generated by five LLMs (Grok-4, Gemini−2.5-pro, ChatGPT-5-2025-08-07, Deepseek-r1-0528, and Claude-opus-4-20250514) were compared with expert-authored plans. A panel of 6 senior physiatrists evaluated the plans using a multi-dimensional framework covering four key domains: Clinical Applicability and Safety (primary safety endpoint), Scientific Rigor, Individualization, and Clarity. To address the data’s hierarchical structure, we employed Linear Mixed-Effects Models (LMM) with random intercepts for cases and raters, and fixed effects for models and language. Pairwise comparisons were adjusted using the Holm-Bonferroni correction.

**Results:**

Quantitative analysis revealed that Grok-4 (mean 4.31) and Gemini−2.5-pro (mean 4.14) significantly outperformed the human benchmark (derived from standardized expert solutions) (mean 3.56; $P<0.001$). Notably, the open-source Deepseek-r1 (mean 3.69) also achieved a statistically significant advantage over experts ($P<0.001$). Conversely, human experts scored numerically higher than Claude-opus-4 (mean 3.50), though this difference was not statistically significant ($P=0.099$). Qualitative analysis further highlighted human experts’ distinct strengths in strategic pathway design and humanistic care.

**Conclusions:**

Top-tier LLMs demonstrate capability in generating high-quality, evidence-based plans, positioning them as effective “executors” for drafting preliminary regimens. We propose a human-AI collaboration paradigm where experts function as “strategists,” focusing on optimization and humanistic care to elevate rehabilitation service quality.

## Introduction

In recent years, the confluence of global population aging and a rising incidence of chronic diseases has led to a surge in demand for rehabilitation services. However, a shortage of specialized professionals in the field has exacerbated the disparity between healthcare service supply and demand [1–3]. Against this backdrop, Artificial Intelligence (AI) technologies, spearheaded by LLMs, are penetrating various domains of healthcare with unprecedented depth and breadth, demonstrating immense potential to reshape clinical practice [4–6]. LLMs can process and synthesize vast amounts of medical literature, clinical guidelines, and patient data [7, 8], showing significant promise in applications such as medical education, physician-patient communication, and clinical decision support [9–11]. Within the field of rehabilitation medicine, preliminary exploratory studies have begun to validate the potential of LLMs. For instance, they can effectively answer common patient questions regarding specific conditions, such as shoulder instability [12], and can assist in drafting initial rehabilitation prescriptions and even interpreting International Classification of Functioning, Disability and Health (ICF) codes [13]. These initial applications present new possibilities for leveraging AI technology to alleviate the strain on rehabilitation healthcare resources.

Despite the promising applications of LLMs, their application evaluation in real-world clinical settings remains in a preliminary stage [14, 15]. The limitations of existing research have led to scholarly skepticism regarding their actual capabilities and reliability in complex clinical tasks. A systematic review published in the *Journal of the American Medical Association* (JAMA) summarized the current state of evaluation in this field, highlighting several key limitations of contemporary research. First, a significant gap exists between prevailing research methodologies and authentic clinical environments. The review found that only a small fraction of studies (approximately 5%) utilized genuine patient data, whereas a majority of evaluations (44.5%) relied on questions from medical licensing examinations, a practice that constrains the generalizability of the findings to actual clinical practice. Second, the dimensions of evaluation require broadening. An overwhelming 95.4% of studies adopted “accuracy” as the primary evaluation metric, while other dimensions crucial for clinical decision-making—such as safety, evidence-based support, level of personalization, and ethical considerations—have not been adequately explored. Finally, research in specific disciplinary areas is insufficient and underrepresented. Physical Medicine and Rehabilitation has received limited attention in published research, accounting for only 0.4% of the literature analyzed [16]. These methodological shortcomings have become a prevailing consensus in the field, with the academic community widely calling for the establishment of more comprehensive, rigorous, and clinically relevant evaluation frameworks. Such frameworks are needed to ensure that assessments of LLMs accurately reflect their real-world value and risks [9, 17, 18].

Concurrently, a focal point of frontier research involves the horizontal performance benchmarking of different LLMs [19–21]. Such studies provide an important means of gauging technological progress by evaluating the performance differences among various types of LLMs. However, while demonstrating and benchmarking the technical capabilities of AI is vital, the ultimate benchmark for evaluating clinical AI is not another AI model, but rather the high level of clinical decision-making ability demonstrated by human experts. Existing LLMs evaluation studies are either based on fictitious cases or lack a direct benchmark against this high standard of expert performance. Consequently, there remains a lack of research in the academic community that directly and systematically benchmarks the performance of these emerging LLMs against that of human experts in the core task of formulating complex rehabilitation plans. The importance of this human-centered [22] evaluation paradigm, which aligns with the prevailing view in the field that AI should be regarded as an “assistive tool for human experts rather than a replacement,” is self-evident [2, 17, 23]. Specifically, we envision the LLM integrated into the clinical workflow as a decision support system that drafts preliminary rehabilitation plans for physician review, thereby streamlining the planning process. Conducting such research is an indispensable step toward objectively understanding the current capability boundaries of AI, uncovering its true clinical value, and exploring future models of human–machine collaboration.

To fill the existing research gap, we designed and implemented a rigorous, blinded evaluation. This study systematically compares the performance of five state-of-the-art LLMs with that of senior rehabilitation medicine experts in formulating clinical rehabilitation plans. We meticulously selected 48 authentic and representative clinical cases from the *Selected Cases in Rehabilitation Medicine from Chinese Clinical Cases series* (2025 publication) [24–29], which were evenly distributed across six rehabilitation subspecialties. Crucially, the human expert benchmark used in this study consists of peer-reviewed solutions from these authoritative texts, representing a “standardized expert reference” of clinical reasoning distinct from the variability of routine daily practice. The evaluation utilized a multi-dimensional quantitative framework co-designed by clinical rehabilitation experts, comprising four core dimensions. To validate the robustness of the findings, all assessments were conducted in independent Chinese and English bilingual environments. Building upon this quantitative assessment, we also conducted a qualitative thematic analysis to deeply investigate the underlying clinical strategies and cognitive traits reflected in the plans from cases where human experts demonstrated superior performance. The core objective of this study is to deeply quantify and analyze the strengths and weaknesses of the most advanced LLMs by directly benchmarking them against plans formulated by human experts. This research not only aims to provide empirical evidence for their responsible application in the field of rehabilitation medicine but also seeks to explore a new paradigm of AI-assisted human-computer collaboration, with the ultimate goal of enhancing the quality and efficiency of rehabilitation healthcare.

**Fig. 1:**
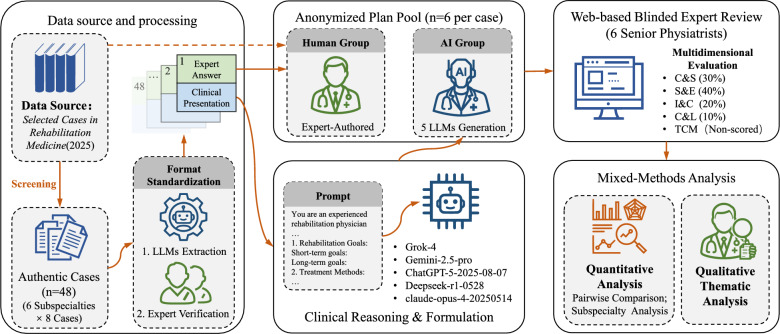
The methodological workflow of the study. The process consists of four phases: (1) Data Preparation: Selection of 48 authentic cases from 6 subspecialties and standardization using Gemini−2.5-pro; (2) Plan Generation: Zero-shot generation of rehabilitation plans by 5 LLMs (Grok-4, Gemini−2.5-pro, ChatGPT-5, DeepSeek-r1, Claude-opus-4) and retrieval of the expert benchmark; (3) Blinded Review: Evaluation by 6 senior physiatrists using a standardized online platform; and (4) Multidimensional Evaluation: Analysis based on four quantitative dimensions and qualitative thematic analysis

## Methods

To systematically investigate the extent to which LLMs can simulate, and even reshape, the complex process of clinical reasoning, we designed a comprehensive experimental study. The overall research framework, illustrating the complete pipeline from data curation to multi-dimensional evaluation, is visually summarized in Fig. 1.

The core design of this study employs a comprehensive evaluation strategy utilizing a high-fidelity bilingual dataset (Chinese and English). This approach allows for a robust estimation of overall model performance via a combined analysis (using LMM), while stratified assessments in each linguistic environment serve to validate the cross-cultural consistency of our findings.

### Data source and processing

To ensure clinical authenticity and authoritativeness, the evaluation dataset was sourced from the *Selected Cases in Rehabilitation Medicine from Chinese Clinical Cases series* (2025 publication), a collection that compiles the clinical experience of nearly 300 front-line experts and closely mirrors real-world practice in China. From an initial pool of 194 cases, our team of rehabilitation experts conducted a rigorous screening process to construct a high-quality evaluation dataset, ultimately selecting 48 authentic cases. The selection was guided by three strict criteria: information completeness, requiring comprehensive patient demographics, medical history, and examination findings; plan completeness, ensuring each case included a detailed and well-structured treatment strategy; and text-centricity. This third criterion was crucial, as it excluded cases heavily reliant on imaging data (e.g., X-rays, MRI) or specific laboratory results for core decision-making to ensure the evaluation focused purely on the models’ ability to comprehend and reason based on textual information. The selected cases were evenly distributed across six major rehabilitation subspecialties—neurological, orthopedic, oncology, pelvic floor, swallowing, and visceral rehabilitation—with eight cases per area, ensuring a balanced and robust foundation for our analysis.

To rule out data contamination and ensure models were not merely retrieving memorized text, we conducted a “Zero-shot Completion Test” prior to evaluation. We prompted all models to complete random text segments (100 200 characters) extracted from 20% of the cases. No model reproduced the original text verbatim; while medically coherent, the outputs exhibited significant divergence in phrasing and structure. This empirically confirms that the models relied on probabilistic clinical reasoning rather than specific pattern retrieval.

To facilitate a standardized and fair evaluation, we then utilized the Gemini−2.5-pro large model—selected for its state-of-the-art text processing capabilities as evidenced by its top-tier ranking on the LMSYS Chatbot Arena leaderboard [30] during the study period—to perform information extraction and formatting on these 48 cases, generating “Clinical Presentation” as uniform model input and structuring the original “Expert Answer” as the human performance benchmark. Crucially, we defined this “Expert Answer” not as a reflection of average routine clinical practice, but as a “standardized expert reference”. These answers consist of peer-reviewed, high-quality treatment exemplars sourced directly from authoritative medical texts, representing an idealized level of clinical reasoning that minimizes the variability typically found in daily practice. To verify the robustness of our findings in a cross-lingual context, we further employed Gemini−2.5-pro to translate all 48 Chinese “Clinical Presentation” and “Expert Answer” documents into English. The accuracy and readability of these translations were subsequently validated, a process that resulted in an English parallel corpus with content identical to its Chinese counterpart for use in supplementary validation experiments. Finally, to minimize potential model-induced biases and ensure the accuracy of the data standardization, all content generated by Gemini−2.5-pro was manually verified item-by-item by our rehabilitation medicine team. During this process, any phrasing exhibiting model-specific stylistic idiosyncrasies was corrected to ensure that the final input text for all models consisted exclusively of objective, standardized clinical facts, confirming that all key clinical information was extracted accurately and completely.

Following this comprehensive process, we constructed a unique, high-fidelity bilingual evaluation dataset. This final dataset consists of 48 corresponding “case-answer” pairs, each containing detailed patient information and an expert-authored treatment plan, available in both the original Chinese and a validated English parallel corpus, providing a solid foundation for the multi-dimensional assessment conducted in this study.

### Evaluated models and plan generation

This study selected five mainstream LLMs, which are widely representative in both industry and academia as of August 8, 2025, as the subjects for evaluation. The selection encompasses leading models from different technical approaches and development institutions:

1. Gemini−2.5-pro [31]
2. ChatGPT-5-2025-08-07 [32]
3. Deepseek-r1-0528 [33]
4. claude-opus-4-20250514 [34]
5. Grok4 [35]

To ensure strict comparability, all models were accessed via their official APIs or web interfaces on a unified date of August 10, 2025. The inference parameter for all models was uniformly set to a temperature of 0.7 to balance generation diversity with stability. All models were utilized in their standard, pre-trained states. No task-specific fine-tuning or additional training on clinical datasets was performed for this study. The evaluation employed a zero-shot inference approach, relying solely on the models’ inherent knowledge bases.

To ensure consistency and fairness in the generation process, we designed standardized prompts in both Chinese and English. The prompt instructed the models to assume the role of a “senior physiatrist” and, based strictly on the provided clinical presentation, formulate a structured plan comprising two sections: “Rehabilitation Goals” and “Therapeutic Methods.” We explicitly stipulated that the models’ analysis and recommendations must be based solely on the patient’s condition as presented in the case files and prohibited the output of any extraneous conversational text to simulate a realistic clinical decision-making scenario. The complete English version of the prompt can be found in Appendix A.

By inputting the 48 Chinese cases and the 48 English cases into each of the five models, respectively, we obtained a total of 480 model-generated rehabilitation treatment plans for the subsequent blinded expert evaluation.

**Table 1:** Scoring dimensions and weight allocation table

Dimension	Description	Weight (%)
Clinical Applicability and Safety (C&S)	Assesses whether the plan conforms to standard clinical practice, is safe and feasible, and is free of critical omissions or contraindications.	30
Scientific Rigor and Evidence-Based Conformity (S&E)	Assesses whether the plan is based on current medical evidence and rehabilitation guidelines, and whether its theoretical basis is scientific.	40
Individualization and Clinical Problem-Solving (I&C)	Assesses whether the plan adequately considers the patient’s specific circumstances (e.g., age, comorbidities, functional status) and is sufficiently targeted.	20
Clarity and Logicality (C&L)	Assesses whether the structure of the plan is clear, the language is professional and precise, and the logical hierarchy is well-defined.	10

1 = Very Poor; 2 = Poor; 3 = Fair; 4 = Good; 5 = Excellent

### Evaluation dimensions

The 5-point Likert scale quantitative evaluation system, which we co-designed with rehabilitation experts, is intended not merely for scoring but, more importantly, to serve as a systematic deconstruction of the cognitive traits that constitute excellent clinical reasoning. This system aims to quantitatively assess the comprehensive quality of rehabilitation plans from multiple core clinical perspectives, with each dimension corresponding to a critical clinical reasoning capability. Notably, the rehabilitation experts who participated in designing the quantitative evaluation system did not take part in the subsequent evaluation and scoring process, thereby ensuring the objectivity of our study. The detailed quantitative evaluation system is presented in Appendix B.

The scoring dimensions and weight allocations are shown in Table 1. The specific weight distribution was established via a Modified Delphi Process by an independent expert panel. Since standardized guidelines for LLM rehabilitation plans are unavailable, this scheme operationalizes a hierarchy of clinical priorities derived from authoritative literature. It prioritizes evidence-based accuracy to mitigate the risk of “hallucinations” (aligned with EBM principles [2, 16]) and adherence to safety norms (per DECIDE-AI guidelines [36]), while explicitly integrating the person-centered functional focus of the ICF framework [22]. This theoretical framework was validated through pilot evaluation to ensure robustness.

In accordance with clinical evaluation standards, we specified the Weighted Total Score as the primary outcome measure to reflect the overall quality of the rehabilitation plans. The Scientific Rigor and Evidence-Based Conformity (S&E) dimension, assigned the highest weight (40%), serves as the core driver for assessing clinical excellence and adherence to evidence-based guidelines. However, distinct from quality assessment, the Clinical Applicability and Safety (C&S) dimension was designated as the independent primary safety endpoint. We explicitly defined a score of $\le 2$ on the C&S dimension as a “significant clinical error” or “safety incident.” Such scores indicate plans containing contraindications or critical omissions that could pose potential risks to patient safety, regardless of their performance in other dimensions.

In addition to the four core scoring dimensions mentioned above, we introduced a non-scored, supplementary dimension specifically for the Chinese evaluation group: Traditional Chinese Medicine (TCM) Design. The purpose of establishing this dimension was to serve as a “stress test,” exploring the adaptability, flexibility, and cultural integration capacity of the AI’s cognitive model when confronted with a fundamentally different medical theoretical system. In the Chinese healthcare system, where the human experts in this study practice, ’Integrative Rehabilitation’—which organically combines modern rehabilitation techniques with TCM modalities like acupuncture and Tuina—is a standard clinical pathway. This is crucial for understanding both the potential and the limitations of AI in reshaping clinical practices that integrate global and local elements.

In the evaluation process for the English-speaking group, the experts used the identical four core dimensions and weighting scheme for scoring but did not include the supplementary TCM Design dimension. This was done to ensure the consistency of the core evaluation framework across both groups.

Through this multi-dimensional, weighted evaluation system, we were able not only to conduct a fine-grained quantitative analysis of the comprehensive quality and specific sub-competencies of the plans generated by each model but also to undertake a preliminary exploration of their adaptability and breadth of knowledge within a specific cultural context.

### Blinded expert evaluation platform and process

Upholding the authority and validity of the benchmark, we adhered to a rigorous purposive sampling strategy to recruit the expert panel. The recruitment was targeted at Grade-A Tertiary Hospitals and leading academic institutions in rehabilitation medicine. The selection criteria for the expert panel were established as follows:1)Educational Background: Possession of a Ph.D. degree in Rehabilitation Medicine or Rehabilitation Therapeutics. 2)Clinical Experience: A minimum of 5 years of active clinical practice in a specialized rehabilitation department. 3)Professional Diversity: The panel must include a multidisciplinary mix of Physiatrists, Physical Therapists (PT), and Occupational Therapists (OT) to reflect the holistic nature of rehabilitation planning. 4)Linguistic Proficiency (Specific to English Evaluation Group): Experts assigned to the English evaluation group must possess a complete English-medium academic background (e.g., overseas doctoral degrees or long-term visiting scholar experiences) to ensure accurate comprehension of English medical terminology and cultural nuances.

With the aim of maximizing inter-rater reliability and consistency, a standardized training session was conducted for all participating experts prior to the formal evaluation. This session covered the operational workflow of the online platform and provided a detailed interpretation of the multi-dimensional scoring rubric. Through a pilot scoring exercise on sample cases, the experts unified their understanding of the evaluation standards to minimize subjective variance.

To ensure the objectivity and professionalism of the evaluation, we developed and deployed a secure, encrypted online platform based on the Python Flask framework. This platform was designed to facilitate remote online assessment by experts from various institutions, employing an evaluation methodology that combines blinded review with multi-dimensional scoring. The online evaluation platform is shown in Appendix C.

Upon logging into the platform, the system sequentially presents a case file. For each case, the platform simultaneously displays six anonymized rehabilitation treatment plans (comprising five plans from the different models and one “Expert Answer” from the book series). To minimize order bias to the greatest extent possible, the platform utilizes a computer-generated random sequence to independently reorder the presentation of the six treatment plans for each evaluator and each case. The evaluators, unaware of each plan’s origin, independently score each one according to the evaluation rubric. This design effectively eliminates brand bias and subjective preferences, ensuring a fair and impartial comparison between the model-generated plans and the expert-authored plan under identical conditions. The formal blinded expert evaluation was conducted over a four-week period from August 20, 2025, to September 14, 2025.

### Statistical analysis

Statistical analyses were performed using Python (version 3.10) with the SciPy and Pingouin libraries. Continuous variables (evaluation scores) are presented as means. To robustly assess the inter-rater reliability of the expert evaluation, we employed a two-step approach. First, to mitigate the influence of individual variations in scoring strictness (leniency/severity bias), raw scores were standardized using Z-score normalization within each rater prior to calculating the Intraclass Correlation Coefficient (ICC) based on a two-way mixed-effects model (ICC 3,k). Second, given that ICC can be sensitive to low variance among high-performing models, we additionally calculated Kendall’s coefficient of concordance (Kendall’s W) on the raw data to evaluate the consistency of the rankings provided by the experts. For the Chinese evaluation group ($k=4$), *W* was derived from the Friedman statistic. For the English evaluation group ($k=2$), *W* was calculated via a linear transformation of Spearman’s rank correlation coefficient ($\rho$) using the formula $W = (1 + \rho )/2$. A P-value $<0.05$ was considered statistically significant.

To rigorously compare the performance of LLMs against human experts while accounting for the hierarchical structure of the data (48 cases evaluated by multiple raters across two language contexts), we employed Linear Mixed-Effects Models (LMM). The weighted total score was modeled as the dependent variable. The model included the “Evaluator” (Specific LLMs vs. Human Expert) and “Language” (Chinese vs. English) as Fixed Effects to assess performance differences and cross-lingual consistency. To account for nested observations and repeated measures, we included random intercepts for both “Case” (Case ID) and “Rater” (Expert ID). For pairwise comparisons between models and the human benchmark, we utilized the estimated marginal means from the LMM, incorporating the Holm-Bonferroni correction to adjust for multiplicity and control the family-wise error rate. A two-tailed adjusted P-value of $<0.05$ was considered statistically significant.

**Table 2:** Inter-rater reliability analysis (ICC and Kendall’s W)

Dimension	Chinese evaluation group (n = 4)	English evaluation group (n = 2)	Combined (n = 6)
C&S	0.66 [0.59, 0.72]*** (W=0.20***)	0.39 [0.23, 0.52]*** (W=0.63***)	0.69 [0.63, 0.74]***
S&E	0.51 [0.41, 0.60]*** (W=0.18***)	0.46 [0.32, 0.57]*** (W=0.65***)	0.57 [0.49, 0.64]***
I&C	0.60 [0.52, 0.67]*** (W=0.28***)	0.27 [0.08, 0.42]** (W=0.57*)	0.63 [0.56, 0.69]***
C&L	0.48 [0.37, 0.57]*** (W=0.20***)	0.49 [0.35, 0.59]*** (W=0.66***)	0.63 [0.55, 0.69]***
TCM	0.86 [0.83, 0.89]*** (W=0.66***)	N/A	N/A
Total Score	0.71 [0.65, 0.76]*** (W=0.23***)	0.54 [0.42, 0.63]*** (W=0.69***)	0.75 [0.70, 0.79]***

Data are presented as ICC (3,k) [95% CI] based on Z-score standardized ratings, and Kendall’s W based on raw rankings

C&S: Clinical Applicability and Safety; S&E: Scientific Rigor and Evidence-Based Conformity; I&C: Individualization and Clinical Problem-Solving; C&L: Clarity and Logicality; TCM: Traditional Chinese Medicine Design

Significance levels: * $$p <0.05$$

** $$p <0.01$$

*** $$p <0.001$$

### Qualitative in-depth analysis

In addition to the quantitative evaluation, a qualitative analysis was conducted to explore the nuanced differences between the rehabilitation plans generated by the top-performing LLMs and those formulated by human experts. This qualitative component of the study was specifically designed to identify and analyze the subtle, yet clinically significant, aspects of rehabilitation planning that may not be fully captured by quantitative metrics.

For this analysis, a purposive sample of 12 clinical cases was selected from the total dataset. This subset comprised instances where the rehabilitation plans authored by human experts received a higher score than the plans generated by the top-performing AI model (determined post-hoc from the quantitative evaluation). This purposive sampling strategy allowed for a focused examination of the factors contributing to the superior performance of human experts in those particular scenarios.

A panel of senior physiatrists, separate from the initial scoring panel, was convened to conduct a comparative thematic analysis of these selected pairs of rehabilitation plans. The physiatrists were tasked with a side-by-side review of the human-generated plans and those generated by the highest-scoring AI model for each selected case. The core objective of this analysis was to deconstruct the qualitative elements that underpinned the superior ratings of the human-authored plans.

Thematic analysis was chosen as the methodological framework for this qualitative inquiry due to its flexibility and suitability for identifying, analyzing, and reporting patterns within textual data. The analysis followed an inductive approach, where themes were allowed to emerge from the data rather than being predetermined. The physiatrist panel engaged in an iterative process of reading and re-reading the plans, identifying initial codes, and subsequently grouping these codes into broader themes that encapsulated the key qualitative differences. Through discussion and consensus-building, the panel refined these themes to ensure they accurately reflected the data. Specifically, three senior physiatrists first coded the cases independently to ensure reliability. Discrepancies were subsequently resolved through a group consensus meeting. We confirmed that data saturation was reached within the purposive sample of 12 cases, as no new distinct themes emerged during the analysis of the final cases. This process aimed to identify the specific qualitative factors that contributed to the perceived superiority of the human-generated rehabilitation plans in the selected cases, thereby providing a deeper understanding of the unique strengths that human experts bring to the rehabilitation planning process.

This study employed a mixed-methods design. The overall clinical evaluation of the AI systems was conducted and reported in strict accordance with the DECIDE-AI (Developmental and Exploratory Clinical Investigations of DEcision support systems driven by Artificial Intelligence) [36] guidelines. The qualitative component, involving the thematic analysis of rehabilitation plans, was reported adhering to the SRQR (Standards for Reporting Qualitative Research) [37, 38] guidelines to ensure methodological rigor and transparency.

## Results

### Baseline characteristics

The evaluation included 48 authentic clinical cases. To ensure a comprehensive evaluation of the models’ capabilities across different domains, these cases were evenly distributed across six major rehabilitation subspecialties: neurological, orthopedic, oncology, pelvic floor, swallowing, and visceral rehabilitation (8 cases per subspecialty). Due to the rigorous screening criteria employed during data selection, data completeness for all cases was 100%, with no missing values in key clinical fields.

The expert panel comprised 6 highly qualified rehabilitation professionals, ensuring a robust human benchmark. Demographic and professional characteristics of the experts are summarized below:1)Demographics and Education: The panel consisted of 3 males and 3 females. Notably, 100% (6/6) of the experts held Ph.D., representing the highest level of academic training in the field. 2)Clinical Experience: The panel demonstrated extensive clinical expertise, with 83.3% (5/6) of experts possessing more than 10 years of experience. Specifically, 2 experts had over 20 years of experience, 3 had 11 20 years, and 1 had 5 10 years of experience. 3)Professional Titles: The hierarchy of professional titles was well-balanced, including 2 Senior (Professor/Chief), 2 Associate Senior, and 2 Intermediate/Junior titles, covering different stages of professional development. 4)Specialization: The panel covered a wide range of subspecialties required for the cases in this study. The experts’ primary clinical foci included neurological(n=5), orthopedic(n=3), oncology(n=1), pelvic floor(n=1), swallowing(n=1), and visceral rehabilitation(n=1) (Note: experts often possess overlapping specialties). 5)Institutional Background: The experts were recruited from high-level institutions, with 5 experts practicing in Grade-A Tertiary Hospitals and 1 expert from a key Medical University, ensuring that the “Human Expert” benchmark reflects top-tier clinical standards.

Regarding the implementation of the evaluation, strict adherence to the study protocol was observed. All 6 invited experts (four from the Chinese assessment group and two from the English assessment group) successfully completed the blinded review of all assigned cases within the specified timeframe, achieving a 100% completion rate. The online evaluation platform maintained stable operation throughout the study period, and no technical interruptions or unblinding incidents were reported. Throughout the evaluation, the versions and parameters of all five LLMs remained locked, with no modifications or updates made to the AI systems. Consequently, we obtained six complete and valid sets of expert ratings, encompassing a total of 1,728 individual plan evaluations (288 unique plans 6 expert evaluations). This comprehensive dataset provides a robust foundation for decoding the performance and mechanisms of AI in reshaping clinical reasoning.

**Fig. 2:**
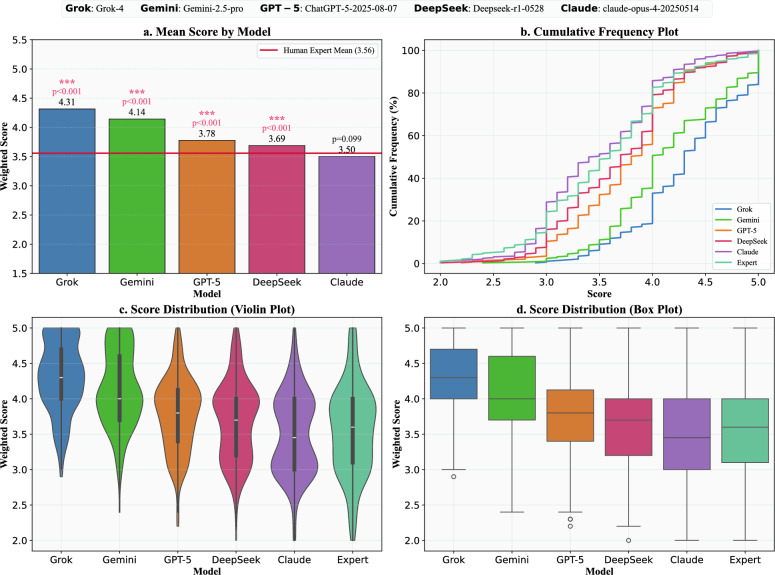
Comprehensive Performance, Stability, and Risk Analysis of LLMs versus Human Experts. **a** Mean Score by Model: This panel displays the average weighted score for each of the five LLMs and the human expert benchmark (indicated by the red horizontal line at $\mu =3.65$). The p-values, derived from a Wilcoxon signed-rank test against the expert benchmark, assess the statistical significance of each model’s performance advantage. **b** Cumulative Frequency Plot: This plot illustrates the cumulative distribution of scores for each evaluator. It shows the percentage of plans that scored at or below a given quality level, providing a tool for assessing “downside risk.” **c** Score Distribution (Violin Plot): This panel visualizes the distribution and probability density of scores for each model and the expert. The width of the violin at any given score level represents the frequency of that score. **d** Score Distribution (Box Plot): Complementing the violin plot, this panel provides a summary of the score distributions, displaying the median, interquartile range (25th to 75th percentile), and outliers

### Quantitative performance evaluation

#### Inter-rater reliability analysis

The results of the reliability analysis are presented in Table 2. The reliability of the expert evaluations was confirmed through the ICC analysis on Z-score standardized data. The weighted total score for the combined dataset achieved a final ICC value of 0.75 [95% CI 0.70 to −0.79], indicating a “good” level of overall reliability ($P <0.001$). This confirms that, despite individual differences in scoring scales, the expert panel maintained a consistent standard for distinguishing model quality tiers.

A nuanced pattern emerged when comparing the two linguistic environments. The Chinese evaluation group demonstrated “moderate-to-good" consistency in scoring across all dimensions ($P <0.001$), with an exceptional consensus on the “TCM Design” dimension ($ICC = 0.86$, 95% CI 0.83 to −0.89, $P <0.001$). Furthermore, the Kendall’s W for TCM was 0.66 ($P <0.001$), indicating strong agreement on both absolute scores and relative rankings.

In contrast, the English evaluation group exhibited a different pattern: a “moderate” ICC for the total score (0.54, $P <0.001$) but a high Kendall’s W (0.69, $P <0.001$). Notably, the I&C dimension in the English group showed the lowest consistency ($ICC = 0.27$, $P =0.004$) and a Kendall’s W of 0.57 ($P =0.013$). While still statistically significant, this reduced agreement suggests that English-speaking experts had the most divergent views when evaluating the personalized aspects of the rehabilitation plans compared to other dimensions. However, for the weighted total score, the high Kendall’s W (0.69) confirms that the relative ranking of the models remained highly consistent between the experts.

#### Overall performance evaluation

This section aims to evaluate the overall performance of each model on the task of formulating rehabilitation treatment plans. We first establish the overall capability of AI by comparing the models’ performance against the human expert benchmark. Subsequently, through pairwise comparisons between the models, we construct a precise performance hierarchy. Finally, we delve into the six major rehabilitation subspecialties to analyze the specificity of each model’s performance.

We compared the overall performance gap between the large models and the human expert benchmark, while also conducting pairwise comparisons among the models. The overall performance evaluation, as shown in Fig. 2a, indicates that most participating LLMs met or surpassed the average performance level of human experts ($\mu =3.56$).

The results of the Linear Mixed-Effects Model analysis, presented in Table 3, reveal a distinct performance hierarchy. Grok-4 (4.31) and Gemini−2.5-pro (4.14) form the top tier, significantly outperforming the human benchmark ($P<0.001$). Notably, the open-source model DeepSeek-r1-0528 (3.69) also demonstrated a statistically significant advantage over human experts ($P<0.001$).In contrast to previous findings, the human expert benchmark (3.56) numerically outperformed Claude-opus-4-20250514 (3.50), although this difference did not reach statistical significance ($P=0.099$). This suggests that when accounting for cross-lingual variability and hierarchical data structure, human experts maintain a competitive edge against current mid-tier models.

**Table 3:** Significance table for overall performance comparison

Evaluator 1	Evaluator 2	Significance	SSP
Gemini	Claude	***	Gemini
Gemini	Deepseek	***	Gemini
Gemini	GPT	***	Gemini
Gemini	Grok	***	Grok
Claude	Deepseek	**	Deepseek
Claude	GPT	***	GPT
Claude	Grok	***	Grok
Deepseek	GPT	*	GPT
Deepseek	Grok	***	Grok
ChatGPT	Grok	***	Grok

In the table, “Gemini” represents the Gemini−2.5-pro model, “Claude” represents the Claude-opus-4-20250514 model, “Deepseek” represents the Deepseek-r1-0528 model, “GPT” represents the ChatGPT-5-2025-08-07 model, and “Grok” represents the Grok4 model. “SSP” stands for Significantly Superior Performer

Significance levels are derived from a LMM with Holm-Bonferroni correction: * $p <0.05$

** $$p <0.01$$

*** $$p <0.001$$

**Fig. 3:**
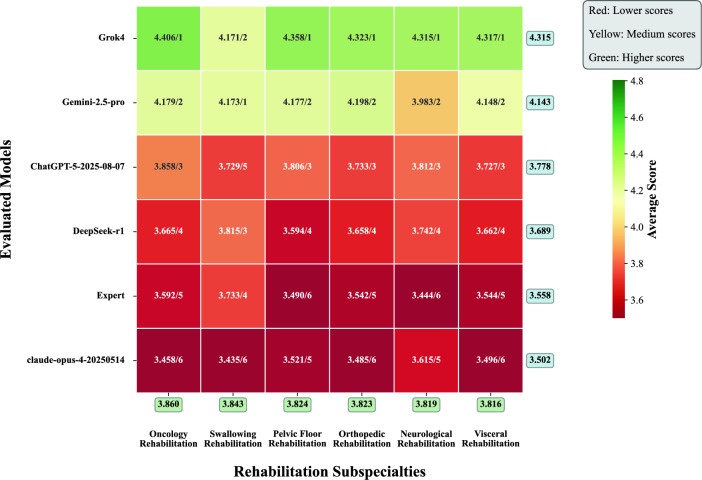
Heatmap of Performance Scores by Rehabilitation Subspecialty.(Format: Score/Rank) The heatmap illustrates a comparative performance analysis of five LLMs and the human expert benchmark across six distinct subspecialties in rehabilitation. Each cell contains two values: the mean performance score and the corresponding rank (formatted as “Score/Rank”). The ranking is determined vertically within each subspecialty column, comparing all evaluated models and the expert. The cell color corresponds to the magnitude of the mean score, with a gradient from red (indicating lower performance) to green (indicating higher performance), as detailed in the color bar. The rightmost column presents the overall average score for each model across all six subspecialties, while the bottom row displays the average performance score for each subspecialty across all evaluated entities, reflecting the relative difficulty of the tasks

To investigate whether model performance exhibits domain specificity, we analyzed the results across the six major rehabilitation subspecialties. The data, as shown in the heatmap in Fig. 3, reveal several key findings. The top-tier models, Grok-4 and Gemini−2.5-pro, demonstrated a pervasive advantage by maintaining a leading position across almost all subspecialties. The analysis also highlighted variations in difficulty among these fields. Human experts showed variable performance across subspecialties, ranking as high as 4th in Pelvic Floor Rehabilitation, but ranking lower in Neurological and Visceral Rehabilitation. Notably, the open-source model DeepSeek-r1-0528 ranked consistently in the middle tier (Rank 3-4 across most specialties), further validating its utility as a reliable, cost-effective clinical support tool.

**Table 4:** Multi-dimensional performance ranking data of the models (Combined Chinese & English Data)

Evaluator	Total score	C&S	S&E	I&C	C&L	TCM
Grok4	4.31/1	4.46/1	4.22/1	4.32/1	4.23/1	1.77/2
Gemini-2.5-pro	4.14/2	4.22/2	4.09/2	4.13/2	4.13/2	1.64/4
ChatGPT-5-2025-08-07	3.78/3	3.87/3	3.75/3	3.76/3	3.65/3	1.58/5
Deepseek-r1-0528	3.69/4	3.79/4	3.67/4	3.68/4	3.48/5	1.58/6
Expert	3.56/5	3.56/5	3.59/5	3.49/6	3.58/4	2.31/1
Claude-opus-4-20250514	3.50/6	3.54/6	3.50/6	3.50/5	3.38/6	1.76/3

Scores are presented as “Mean Score/Rank”. “Total Score” represents the weighted average of core dimensions. “TCM” data is derived exclusively from the Chinese dataset as it is a culture-specific dimension. Models are ranked based on the Combined (Chinese + English) dataset performance

**Fig. 4:**
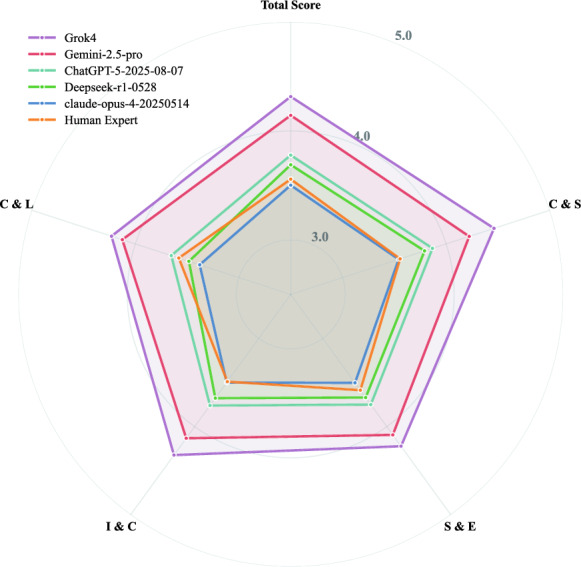
Radar Chart of Multi-dimensional Performance. This radar chart compares the performance of the five evaluated LLMs and the human expert across four core dimensions of clinical reasoning: A larger enclosed area signifies superior overall performance

Furthermore, the performance gap between human experts and the top-tier models was most pronounced in subspecialties requiring the integration of complex, multi-system information, such as “Neurological Rehabilitation” and “Orthopedic Rehabilitation.” This underscores the particular advantage of LLMs in systematic information integration. Notably, the analysis also revealed the significant application potential of open-source models. The open-source model DeepSeek-r1-0528, for example, ranked third in the complex domain of “Neurological Rehabilitation” and outperformed the human expert. This demonstrates that even non-top-tier models can provide high-quality decision support in specific clinical scenarios, indicating the feasibility of cost-effective deployment.

In summary, the subspecialty analysis reveals that the performance of LLMs is not uniform but exhibits significant domain specificity. This provides important clues for their precise clinical application and future optimization.

#### Multi-dimensional performance analysis

To investigate the underlying reasons for the models’ overall performance advantages, we decomposed the scores across the core evaluation dimensions. The analysis reveals a systematic superiority of the top-tier AI models over the human expert benchmark. As detailed in Table 4 and visualized in the radar chart (Fig. 4), this advantage was consistent across most core dimensions of clinical reasoning.

In terms of usability and information presentation, reflected by the C&L dimension, Grok-4 (4.23) and Gemini−2.5-pro (4.13) achieved high scores, suggesting that the AI-generated plans were professionally structured and easily comprehensible for clinicians, requiring minimal cognitive effort to interpret. Notably, human experts (3.58) ranked 4th in this dimension, outperforming both DeepSeek-r1-0528 and Claude-opus-4, indicating that human-authored plans retain a competitive edge in professional readability.

Grok-4 demonstrated consistent excellence across all evaluation metrics, ranking first in every core dimension. Crucially, regarding the designated primary safety endpoint—C&S—Grok-4 achieved a remarkable mean score of 4.46, demonstrating exceptional reliability alongside its therapeutic precision. It is worth noting that human experts (3.56) ranked 5th in this dimension, surpassing Claude-opus-4 (3.54), reflecting the experts’ rigorous adherence to safety protocols. Importantly, high-quality, systematic decision support is not exclusive to top-tier proprietary models. The open-source model, DeepSeek-r1-0528, also consistently surpassed the human expert’s performance across most core dimensions (C&S, S&E, and I&C), indicating that this capability has become a common characteristic of modern mainstream AI.

However, in contrast to their excellent performance on the core dimensions, the supplementary “TCM Design” dimension revealed a distinct pattern. In this context, the performance hierarchy was reversed: human experts ($\mu$=2.31) ranked first, scoring significantly higher than all models, with the top-performing Grok-4 achieving only 1.77. It is important to interpret this result with caution. Since our study design utilized geographically neutral clinical vignettes and did not explicitly mandate the inclusion of TCM elements, the LLMs’ omission of these modalities does not constitute a capability deficit. Instead, it reveals a distinct “Paradigm Divergence.” The LLMs defaulted to a globally dominant Western medical paradigm, providing standard, evidence-based rehabilitation plans applicable worldwide. In contrast, human experts, influenced by their specific clinical environment, spontaneously integrated localized therapeutic methods (TCM) as a complementary approach. This suggests that while LLMs excel at standardization, human experts are more prone to implicit localization based on their professional background.

#### Stability and risk assessment

Beyond average performance, clinical applications demand exceptional stability and reliability in treatment plans. Our analysis of score distributions reveals that the stability of the models is highly correlated with their performance hierarchy. Top-tier models like Grok-4 and Gemini−2.5-pro not only achieved high average scores, but their outputs were also highly concentrated in the high-score range, demonstrating exceptional stability (Fig. 2c, d). This stands in stark contrast to the plans from human experts, which exhibited the broadest score distribution, reflecting considerable variability in clinical opinions and comparatively lower stability.

This difference in stability translates to a quantifiable gap in risk. The cumulative frequency plot (Fig. 2b) provides a powerful tool for assessing this “downside risk.” Using a score of 4.0 as the threshold for high quality, the probability of a top-tier model generating a high-quality plan was more than double that of a human expert (approximately 80% vs. fewer than 40%). Concurrently, their risk of producing moderately to poorly scored responses was significantly lower. In summary, the top-tier LLMs’ advantage extends beyond mean performance to a significant and quantifiable superiority in performance stability and risk control—the ability to consistently produce high-quality responses while effectively avoiding low-scoring ones.

#### Robustness validation of performance comparison

To investigate the stability of model performance across different linguistic and cultural contexts, we performed a stratified analysis of the Chinese and English datasets. Unlike the primary LMM analysis which pooled data to maximize statistical power, this breakdown aims to detect potential inconsistencies or contextual dependencies. The results of the comparison between Chinese and English environments are presented in Table 5.

First, the top-tier status of Grok-4 and Gemini−2.5-pro remained highly robust and consistent across both languages. Their performance advantage was statistically significant in both subsets, reinforcing the validity of the combined analysis.

However, contextual dependencies were observed in the middle tier. For instance, the comparison between Human Experts and Claude-opus-4 was inconsistent; while there was no significant difference in the Chinese context ($P=0.087$), human experts significantly outperformed Claude in the English evaluation ($P<0.001$). This discrepancy suggests that Claude’s performance relative to experts may be sensitive to linguistic nuances.

Conversely, the relationship between DeepSeek-r1-0528 and ChatGPT-5 showed consistency, with no statistically significant difference observed in either linguistic environment under the rigorous LMM analysis. In conclusion, this cross-validation supports that while the superior performance of top-tier LLMs is a highly robust finding, the evaluation outcomes for mid-to-low-tier models and the human expert are subject to context dependency.

**Table 5:** Comparison of the Consistency of Evaluation Results in Chinese and English Environments (Based on LMM Analysis)

Evaluator 1	Evaluator 2	Chinese evaluation		English evaluation		Consistency
		Sig.	SSP	Sig.	SSP	
Expert	Gemini	***	Gemini	***	Gemini	Consistent
Expert	Claude	n.s	None	***	Expert	Inconsistent
Expert	Deepseek	***	Deepseek	n.s	None	Inconsistent
Expert	GPT	***	GPT	*	GPT	Consistent
Expert	Grok	***	Grok	***	Grok	Consistent
Gemini	Claude	***	Gemini	***	Gemini	Consistent
Gemini	Deepseek	***	Gemini	***	Gemini	Consistent
Gemini	GPT	***	Gemini	***	Gemini	Consistent
Gemini	Grok	**	Grok	***	Grok	Consistent
Claude	Deepseek	n.s	None	***	Deepseek	Inconsistent
Claude	GPT	***	GPT	***	GPT	Consistent
Claude	Grok	***	Grok	***	Grok	Consistent
Deepseek	GPT	n.s	None	n.s	None	Consistent
Deepseek	Grok	***	Grok	***	Grok	Consistent
GPT	Grok	***	Grok	***	Grok	Consistent

In the table, “Gemini” represents Gemini−2.5-pro, “Claude” represents Claude-opus-4-20250514, “Deepseek” represents Deepseek-r1-0528, “GPT” represents ChatGPT-5-2025-08-07, and “Grok” represents Grok4. “SSP” stands for Significantly Superior Performer

n.s.: indicates a non-significant result

Significance levels (Sig.) are derived from a LMM with Holm-Bonferroni correction: *$p < 0.05$

**$$p < 0.01$$

***$$p < 0.001$$

### Qualitative in-depth analysis

While our quantitative analysis demonstrates the formidable capability of leading LLMs in formulating high-quality, evidence-based rehabilitation plans, a purely data-driven perspective is insufficient to capture the full spectrum of clinical excellence. Raw scores, while indicative of systematic rigor, can obscure the nuanced, higher-order cognitive traits that define truly exceptional clinical practice. To investigate these attributes, we selected cases where the human expert’s rating was significantly higher than that of the top-performing LLM (Grok-4). A panel of senior physiatrists then conducted a comparative thematic analysis of these cases, tasked with identifying and deconstructing the qualitative factors that underpinned the superiority of the human-authored plans.

Our findings reveal that, in these instances, the superiority of the human expert was not merely incremental but represented a distinct shift in thinking—from a “treatment executor” to a “medical strategist.” This strategic mindset manifested across four key dimensions.

#### Strategic path design

 A fundamental distinction emerged in the architectural design of the rehabilitation plans. The plans generated by the LLMs, while comprehensive, often presented as a static, modular “checklist of therapies.” In contrast, the human expert demonstrated a superior ability to design a dynamic, multi-stage, and interdisciplinary Clinical Pathway that evolves with the patient’s journey.

For instance, in a complex case of dysphagia and aphonia, the human expert structured the intervention into three distinct, theme-based phases (e.g., Phase 1: “Reconstructing the Swallowing Reflex”). All therapeutic modalities—swallowing, physical, vocal, and nursing—were strategically integrated to serve the central goal of each phase, showcasing a clear, time-based progression. Grok-4, conversely, organized its plan by functional domains ("Swallowing Function,” “Motor Function”), which, while logical, lacked this overarching temporal and strategic cohesion.

This strategic foresight was even more pronounced in a cardiac transplant case. The expert’s plan encompassed the entire patient continuum, beginning with pre-weaning interventions in the ICU (e.g., specific exercises on a SIMV ventilator setting) and extending to a detailed follow-up schedule at 3 and 6 months post-discharge. The LLM’s plan focused competently on the inpatient phase but lacked this “whole-lifecycle” management perspective. The human expert, therefore, operated not just as a therapist but as a project manager for the patient’s entire recovery arc.

#### Clinical insights

The human expert consistently demonstrated an exceptional ability to penetrate the complex layers of a clinical presentation to identify and prioritize the “core problem”—the primary limiting factor hindering the patient’s overall progress. This allowed for a highly effective and efficient allocation of therapeutic efforts.

A compelling illustration was a case involving a patient with cancer and hemiplegia who was experiencing severe emotional distress. The human expert made a crucial strategic decision to place the “Psychiatric Intervention” section before the “Rehabilitation Therapy” section, with “Emotional and Sleep Regulation” listed as the primary short-term goal. This strategy reflects a profound clinical judgment that psychological stability was an absolute prerequisite for any physical rehabilitation to be effective. While Grok-4 also provided a detailed psychological plan, it was listed in parallel with other therapies, missing this critical hierarchical insight.

Furthermore, in a pelvic floor dysfunction case, the expert’s diagnostic thinking transcended the musculoskeletal system. By incorporating “visceral manipulation” and techniques for uterine conditioning, the expert addressed the possibility that the dysfunction originated from deeper visceral or fascial restrictions. Grok-4’s plan, though technically proficient in musculoskeletal interventions, did not exhibit this “inside-out” etiological perspective, showcasing the expert’s deeper, more holistic diagnostic acuity.

#### Technical specificity and paradigm synthesis

The human expert’s therapeutic toolkit was not only broader but also demonstrated deeper technical specificity and the ability to seamlessly synthesize different medical paradigms. This was particularly evident in the application of advanced technologies and culturally-specific modalities like TCM design.

The expert’s plans often included high-precision, cutting-edge interventions. For instance, when recommending repetitive transcranial magnetic stimulation (rTMS), the expert specified precise parameters (e.g., 90% intensity, 50Hz frequency), elevating the recommendation from a general suggestion to an executable clinical prescription. In the cardiac transplant case, the expert explicitly detailed the “Sternal Precautions” (the “barrel principle”), a cornerstone of safety in this context that was absent in the LLM’s otherwise thorough plan.

Most notably, this analysis highlights a divergence in paradigm integration between LLMs and human experts. As our study used clinical cases from China, the human experts naturally and proactively integrated TCM based on deep theoretical foundations. In one oncology case, the expert provided a precise TCM syndrome differentiation and prescribed a specific herbal formula and detailed acupuncture techniques. This represents a true “chemical fusion” of two medical systems. This qualitative finding strongly corroborates our quantitative results from the supplementary “TCM Design" dimension, where human experts ($\mu$=2.31) significantly outperformed all LLMs. Given that the prompt did not request TCM, the LLMs’ adherence to a globally dominant Western medical paradigm reflects a tendency towards “Global Standardization.” In contrast, the human expert fluidly navigates and synergizes multiple frameworks to optimize patient care, demonstrating a unique capacity for “Local Contextualization” and implicit cultural adaptation.

#### Humanistic care

Finally, the essence of the human expert’s plans lay in their profound patient-centeredness. The goal was consistently framed not just as restoring function but as empowering the individual to reclaim their life, social roles, and sense of self.

This was achieved through the “contextualization” and “meaning-making” of therapeutic goals. For a stroke patient who was a “fruit shop owner,” the expert designed cognitive training that simulated the real-world tasks of “stocking, arranging, and selling fruit.” This is profoundly more engaging and transferable than the LLM’s standard, decontextualized exercises like “picture recall” or “number sequencing.” Similarly, a functional goal like “independently don and doff a cardigan” is more tangible and motivating for a patient than an abstract metric like “achieve an MBI score of 80.”

Moreover, the expert’s approach was fundamentally empowering. When addressing a patient’s anxiety, the expert went beyond providing counsel and actively taught the patient a lifelong self-management tool: the “Three-Column Record” technique. This philosophy of equipping patients with the skills to manage their own condition stands in contrast to the LLM’s approach, which positions the therapist as the primary agent of change. It is this focus on restoring the patient’s agency and dignity that represents the highest level of humanistic care.

These qualitative findings, juxtaposed with the quantitative results, paint a comprehensive picture of the complementary strengths of AI and human experts, paving the way for a new collaborative paradigm, as we will discuss in the following section.

## Discussion

The core finding of this study is twofold. On one hand, LLMs demonstrate powerful capabilities in the systematic and evidence-based aspects of rehabilitation plan formulation. On the other hand, human rehabilitation specialists possess an indispensable value in higher-order strategic planning and the nuanced dimensions of patient-centered care. This finding directly addresses our primary research objective—to explore the differences between AI and rehabilitation physicians—and offers a clear direction for the future of rehabilitation medicine. Collectively, these findings provide robust empirical evidence supporting the intended use of LLMs as clinical decision support systems. They demonstrate the capability to function as reliable ’drafting assistants’ for preliminary rehabilitation plans, provided they are deployed within a workflow that includes expert human oversight.

Given the multidimensional nature of this evaluation—spanning quantitative metrics, qualitative themes, and cross-cultural comparisons—we adopted a hybrid reporting structure that integrates brief interpretive commentary within the Results section. This approach was chosen to facilitate the immediate contextualization of complex findings and enhance clinical readability.

Our quantitative results, reinforced by the Linear Mixed-Effects Model analysis, provide robust evidence of the potential for LLMs to serve as support tools within rehabilitation medicine. The leading models (specifically Grok-4 and Gemini−2.5-pro) consistently generated systematic, evidence-based treatment plans with a level of consistency, comprehensiveness, and risk mitigation that met or exceeded the benchmark set by human rehabilitation specialists(representing an idealized, standardized level of expertise). A significant finding from the updated analysis is the performance of the open-source model DeepSeek-r1-0528. By statistically outperforming the human benchmark ($P<0.001$) in the combined analysis, it indicates that LLMs can reliably undertake knowledge-intensive foundational tasks, providing a high-quality and efficient starting point for decision-making in rehabilitation.

However, the analysis also revealed that human experts maintained a higher degree of robustness compared to mid-tier models like Claude-opus-4, particularly when data from diverse linguistic contexts were aggregated. This robust performance, alongside the qualitative findings, sharply accentuates the unique value of the rehabilitation specialist’s expertise. Our qualitative analysis demonstrates that high-quality practice in rehabilitation medicine is a higher-order cognitive activity that transcends information processing. These specialists excel at designing dynamic rehabilitation pathways, applying deep insight to prioritize the core problems that determine recovery outcomes, and delivering empowerment-focused, humanistic care. These capabilities—particularly the wisdom to proactively integrate localized medical paradigms to provide context-aware care—constitute the sophisticated dimensions of expertise in rehabilitation that are not readily codifiable and remain challenging for current AI architectures.

Consequently, our research does not lead to a conclusion of AI replacing rehabilitation specialists, but instead clearly defines a new paradigm of Human-AI Collaboration in Rehabilitation. Within this framework, the role of an LLM is not that of a competitor, but as an essential and highly efficient assistant for the human rehabilitation specialist. Our objective is to merge the systematic strengths of AI with the strategic and holistic wisdom of the human specialist to achieve enhanced rehabilitation outcomes.

In this collaborative paradigm, the division of labor becomes clear and complementary. The LLMs assumes the role of a systematic “executor” and “knowledge base,” tasked with knowledge-intensive but procedural work: rapidly retrieving high-volume evidence from clinical guidelines, integrating fragmented quantitative assessment data (e.g., muscle strength grades, range of motion), and synthesizing this information into a comprehensive, standardized draft of the rehabilitation plan. The rehabilitation specialist, liberated from these laborious tasks, can then focus on higher-value work as the ultimate “strategist” and provider of patient-centered care. Their responsibilities are elevated to addressing the nuanced dimensions of clinical reasoning: designing dynamic clinical pathways that evolve with the patient’s condition, prioritizing core limiting factors in complex comorbidities, and managing the crucial human interactions required to build a therapeutic alliance and align treatment goals with the patient’s personal life and dignity. Crucially, this human-in-the-loop oversight serves as a vital safety gate to mitigate the risks of misaligned or incomplete model recommendations, which, if left unverified, could lead to adverse clinical consequences such as inappropriate therapeutic intensity or overlooked contraindications.

It is critical to emphasize that the effectiveness of this collaboration is predicated on the specialist’s robust clinical knowledge base. Rather than diminishing the importance of expertise, the role of a “Strategist” elevates the cognitive threshold for clinicians, who must possess sufficient mastery of rehabilitation principles to critically audit AI-generated drafts, identify nuanced clinical omissions, and challenge recommendations that may not align with complex, real-world patient needs. Without this foundational knowledge, the therapist risks becoming a passive recipient of AI outputs rather than an informed decision-maker.

Notably, our findings regarding high-performance open-source models provide a critical catalyst for the widespread adoption and future development of this collaborative paradigm. The fact that DeepSeek-r1-0528 achieved a statistically significant advantage over human experts in the LMM analysis demonstrates that building effective and accessible AI assistants for rehabilitation is both technically and economically feasible. This lays the groundwork for developing localized models tailored to specific cultures (such as integrating Traditional Chinese Medicine knowledge), thereby allowing this advanced collaborative model to benefit a broader range of regions and populations.

While this study offers significant conclusions, its findings must be interpreted in the context of several inherent limitations:

1. Text-Centricity of the Evaluation: Our assessment was based exclusively on textual case summaries and did not incorporate the multimodal information critical to real-world clinical scenarios, such as medical imaging or video gait analysis. This limits the scope of the evaluation, as the ability to process purely textual information is not entirely equivalent to the comprehensive decision-making capabilities required in a multimodal environment.
2. Potential Bias in Data Standardization: This study utilized Gemini−2.5-pro to extract and format information from the original cases to ensure a uniform input structure for all models. While this standardization was a critical methodological step to facilitate a rigorous, blinded evaluation and prevent order or brand bias, a theoretical possibility exists that the text structure generated by a specific model might be more aligned with its own comprehension paradigm. However, we posit this effect is minimal and manageable, as the task primarily involved highly structured information extraction—a capability where current mainstream LLMs demonstrate high accuracy [39]. Furthermore, all extracted “Expert Answers” underwent item-by-item manual verification by our rehabilitation team to ensure that the core therapeutic measures remained consistent with the original clinical expertise in the source text.
3. Nature of the “Expert Answer” Benchmark: The “Expert Answer” used as the evaluation benchmark was sourced from authoritative textbooks, representing a refined and standardized ideal case. While this provided a high-quality and stable benchmark, such an ideal exemplar may differ from decision-making processes in routine clinical practice, which are often characterized by uncertainty and significant patient variability [40]. Therefore, our findings are more accurately interpreted as top-tier LLMs surpassing an “idealized expert level” rather than being directly equivalent to outperforming the “average expert in routine practice.” Furthermore, while LLMs showed high alignment with these standardized answers, there is a risk of overestimating their performance in complex, non-textbook scenarios where patient symptoms are often ambiguous and data may be missing—conditions where human clinical expertise remains indispensable for navigating uncertainty. Additionally, real-world clinical data often involves heterogeneous formats (e.g., handwritten notes) and potential human errors in documentation, which present both a challenge for LLM integration and an opportunity for future systems to assist in error detection and data standardization.
4. Simulation-Based Study Design: While this study employed authentic cases, it remains a retrospective simulation benchmarking study. The AI systems were evaluated in a controlled, in silico environment rather than in a prospective clinical workflow involving direct patient interaction. Consequently, the study focuses on the theoretical quality of the plans and does not measure actual patient health outcomes or the real-world impact on physician efficiency (e.g., time-saving metrics). Future research should transition to prospective clinical trials to validate these findings in live healthcare settings.
5. Representativeness of the Expert Panel: Although the evaluation panel was composed of senior experts, the sample size was relatively limited. Their scoring criteria may have been influenced by regional factors, schools of thought, or personal clinical experience, and thus may not fully represent the entire spectrum of viewpoints within rehabilitation medicine [41].
6. Rapid Technological Iteration: LLMs technology is evolving at an unprecedented pace. The conclusions of this study are limited to the specific model versions tested at the time of the evaluation. Future models or updated versions may exhibit different performance characteristics.
7. Neutrality of Prompts and TCM Assessment: The lower scores of LLMs in the TCM dimension should be viewed as a reflection of their default behavior under neutral instructions, rather than a lack of knowledge. Our prompts were intentionally designed to be geographically and culturally neutral to simulate a generalized query. Consequently, models provided standard international care regimens. The “TCM Design” dimension therefore serves as an exploratory analysis highlighting the difference between the AI’s “universal” approach and the human expert’s “situated” practice, rather than a metric of core clinical competence.

## Conclusions

In summary, this study provides a clear delineation of the core differences in reasoning for rehabilitation planning between LLMs and human rehabilitation specialists through a comprehensive quantitative and qualitative comparison. We have confirmed that LLMs possess the robust capability for systematic, evidence-based plan formulation required to serve as effective assistants in rehabilitation practice. Simultaneously, we have highlighted the distinct value of rehabilitation physicians in strategic planning, practical insight, and humanistic care. The central conclusion of our research is therefore not about AI replacing human rehabilitation clinicians, but the proposal of a more constructive paradigm of human-AI collaboration. In this model, AI serves as a powerful tool to free rehabilitation specialists from burdensome knowledge-based work, allowing them to concentrate on the more nuanced, strategic, and humanistic dimensions of their practice. We believe that this collaborative approach, which merges the systematic strengths of AI with the depth of the human specialist’s wisdom, is the key path toward a future of higher-quality, more efficient, and more humane rehabilitation care.

## Figures and Tables

**Fig. 5:**
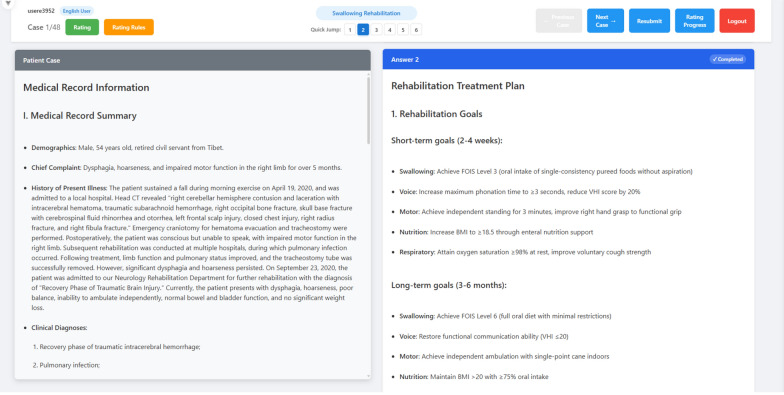
Main interface of the online evaluation platform. This interface displays the case content and the corresponding rehabilitation plan, facilitating blind review and scoring by experts according to the evaluation rules

## Data Availability

The codes and data are available at https://github.com/WenhC09/LLM-Rehab-Benchmark.

## Appendix A

### Full English prompt

The content of the prompt was identical in both the Chinese and English versions. The complete English version is as follows:

## Appendix B

### Evaluation dimensions for large models in rehabilitation

Dimension 1: Clinical Applicability and Safety (Weight: 30%)

*Core Question*: Does the plan adhere to clinical norms, is it safe for the patient, and does it clearly emphasize the most critical safety principles for the current stage?

The detailed scoring rubric for Dimension 1 is shown in Table 6, with its evaluation points listed as follows:

1. * Emphasis on Core Safety Principles*: Does the plan clearly define and emphasize the most critical safety baselines for the current stage (e.g., NPO status, weight-bearing restrictions, activity contraindications)?
2. * Diagnostic Congruence*: Do the therapeutic interventions in the plan correspond precisely with the diagnosis presented in the case?
3. * Contraindication and Risk Mitigation*: Does the plan explicitly identify and exclude the patient’s absolute or relative contraindications, and does it anticipate potential risks?
4. * Clarity and Reasonableness of Parameters*: Are the parameters for treatment—such as intensity, frequency, duration, and load—clearly defined, quantified, reasonable, and operationally feasible?
5. * Stage-Appropriate Safety*: Are the safety measures within the plan appropriate for the patient’s specific rehabilitation stage and environment (e.g., vital sign monitoring during the ICU phase, early postoperative weight-bearing restrictions in orthopedics)?

**Table 6:** Detailed scoring rubric for dimension 1: clinical applicability and safety

Score	Level	Specific scoring criteria
5 points	Excellent	*Perfect Match and Risk Aversion:* The plan is perfectly congruent with the diagnosis, accurately identifies and mitigates all potential contraindications and risks, and explicitly emphasizes core safety principles. *Precise and Clear Parameters:* All therapeutic parameters are extremely specific, quantitative, and highly actionable. *Comprehensive Risk Preparedness:* Provides specific, executable preventive measures and recommendations for handling emergencies relevant to the current stage.
4 points	Good	*High Congruence and Consideration:* The plan is highly relevant to the diagnosis, with major contraindications and risks considered, and no apparent safety flaws. *Reasonable Parameters:* Therapeutic parameters are set reasonably, conform to standard clinical practice, and most instructions are clear. *Strong Risk Awareness:* Mentions major potential risks and provides conventional preventive recommendations.
3 points	Fair	*Basic Match:* The plan is generally applicable to the diagnosis but lacks thorough consideration of certain minor contraindications or potential risks. *Parameters within Safe Range:* Some therapeutic parameters are slightly conservative, aggressive, or vaguely described, but remain within an acceptable safety margin overall. *Insufficient Risk Awareness:* Mentions therapeutic risks in a generalized manner.
2 points	Poor	*Mismatch or Negligence:* The plan includes therapeutic items not applicable to the diagnosis or overlooks obvious clinical contraindications, posing a foreseeable safety hazard. *Inappropriate Parameters:* Therapeutic parameters are set unreasonably or are so vaguely described as to be inexecutable.
1 point	Very Poor	*Severe Violation or Harmful:* The plan contains serious safety issues, directly violates established clinical contraindications, is highly likely to cause direct harm to the patient, or is completely incongruent with the diagnosis.

Dimension 2: Scientific Rigor and Evidence-Based Conformity (Weight: 40%)

*Core Question*: Do the therapeutic methods recommended in the plan align with the current evidence-based consensus and best practices in the field of rehabilitation medicine?

The detailed scoring rubric for Dimension 2 is shown in Table 7, with its evaluation points listed as follows:

1. * Conformity to Evidence-Based Practice*: Based on the expert reviewer’s professional judgment, are the core therapeutic methods recommended in the plan recognized as effective approaches supported by high-level evidence in the current field?
2. * Prioritization of Therapies*: Does the plan prioritize the selection of “first-line” therapies that are most effective for the core problems and are recommended by authoritative guidelines?
3. * Use of Current and Appropriate Techniques*: Does the plan incorporate recognized, current, and effective techniques, while avoiding outdated or disproven methods?
4. * Avoidance of Controversial or Ineffective Treatments*: Does the plan exclude therapeutic methods that lack reliable evidence, are highly controversial regarding their efficacy, or have been proven to be ineffective?

**Table 7:** Detailed Scoring Rubric for Dimension 2: Scientific Rigor and Evidence-Based Conformity

Score	Level	Specific scoring criteria
5 points	Excellent	*Conforms to Best Practices:* All core therapies recommended in the plan are recognized best practices in the current field, supported by high-level evidence (e.g., Grade A recommendations in authoritative guidelines known to the reviewer). *Precise Selection:* The selection of core therapies is highly precise, reflecting a deep understanding of the problem’s nature. *Unadulterated Plan:* Contains no ineffective or controversial treatment recommendations whatsoever.
4 points	Good	*Conforms to Mainstream Consensus:* Most core therapies align with the mainstream evidence-based consensus (e.g., Grade B or higher recommendations in guidelines known to the reviewer). *Reasonable Selection:* The overall selection of therapies is scientific and reasonable. *Largely Uncontroversial:* Includes very few therapies with unclear evidence or existing controversy.
3 points	Fair	*Conforms to Basic Principles:* The plan primarily consists of recognized, standard therapies but may not have selected the most effective methods or those with the highest level of evidence. May include some ancillary therapies with weaker evidence. “*Catch-all” Selection:* May list a wide range of therapies without a clear sense of prioritization.
2 points	Poor	*Questionable Evidence Base:* The plan includes a significant number of therapies that lack reliable evidence or are controversial within the field. *Includes Ineffective Therapies:* Recommends therapies that have been shown by consensus to be ineffective or are considered obsolete.
1 point	Very Poor	*Violates Evidence-Based Principles:* The plan is primarily based on theories that have been disproven, are outdated, or are pseudoscientific, making it seriously misleading.

Dimension 3: Individualization and Clinical Problem-Solving (Weight: 20%)

*Core Question*: Does the plan accurately interpret the quantitative assessment data from the case, identify and prioritize the core problems, and formulate a function-oriented, staged solution?

The detailed scoring rubric for Dimension 3 is shown in Table 8, with its evaluation points listed as follows:

1. * Responsiveness to Key Assessment Data*: Does the plan directly incorporate the quantitative data provided in the case (e.g., muscle strength grades, range of motion, swallowing assessment levels) to guide the treatment strategy?
2. * Problem Identification and Prioritization*: Does the plan successfully identify the most critical functional impairments from the complex clinical presentation and prioritize them for intervention?
3. * Staged and Dynamic Planning*: Does the plan reflect the staged nature of rehabilitation? Does it propose specific criteria for progressing from one stage to the next?
4. * SMART Functional Goal Setting*: Does the plan establish Specific, Measurable, Achievable, Relevant, and Time-bound (SMART) functional goals for the prioritized problems?
5. * Integrative and Interdisciplinary Approach*: Does the plan demonstrate an interdisciplinary approach (e.g., by recommending nutritional consultation, psychological support, etc.)?

**Table 8:** Detailed Scoring Rubric for Dimension 3: Individualization and Clinical Problem-Solving

Score	Level	Specific scoring criteria
5 points	Excellent	*Data-Driven Decision-Making:* Core recommendations are directly linked to and based on specific quantitative data from the case. *Precise Prioritization and Staging:* Clearly identifies and prioritizes the primary rehabilitation problem, proposes a clear, staged rehabilitation pathway, and provides specific criteria for progression. *Precise SMART Goals:* Establishes highly specific, quantifiable SMART functional goals centered on the core problem.
4 points	Good	*Responsive to Key Information:* The plan is clearly designed based on the main clinical features and assessment results from the case. *Major Problems Identified:* Identifies the main functional impairments, but prioritization or staging may not be sufficiently clear. *Clear Functional Goals:* Sets relatively clear and measurable functional goals.
3 points	Fair	*Diagnosis-Based Template:* The plan is primarily based on the medical diagnosis but is insufficiently responsive to the unique assessment data in the case, showing signs of being “template-like.” *Problem Listing:* Lists the patient’s problems but fails to establish clear priorities. *General Goals:* Sets functional goals, but they are not specific or measurable enough.
2 points	Poor	*Highly Template-Based:* The plan is a standardized “disease template” that almost completely ignores the unique quantitative assessment information provided in the case. *Lacks Problem Analysis:* Does not show any analysis of the patient’s core problems. *No Functional Goals:* It is merely a collection of therapeutic items without any functional goals being set.
1 point	Very Poor	*Completely Generic or Mismatched:* The plan is entirely generic, or the recommendations contradict the patient’s assessment results.

Dimension 4: Clarity and Logicality (Weight: 10%)

*Core Question*: Is the presentation of the plan clear and easy to understand? Is its internal logic coherent?

The detailed scoring rubric for Dimension 4 is shown in Table 9, with its evaluation points listed as follows:

1. * Structural Clarity*: Is the organizational structure of the plan logical, well-hierarchized, and easy to read and comprehend?
2. * Professionalism and Readability of Language*: Does it successfully balance the accuracy of professional terminology with comprehensibility for a layperson (such as a patient’s family)?

**Table 9:** Detailed Scoring Rubric for Dimension 4: Clarity and Logicality

Score	Level	Specific scoring criteria
5 points	Excellent	The structure is extremely clear with exceptionally strong logic. The language is precise and easy to understand, balancing professionalism with educational value.
4 points	Good	Clear structure and coherent logic. Highly professional with good readability.
3 points	Fair	The structure is generally reasonable, but there may be some logical gaps. Overuse of technical jargon, with fair readability.
2 points	Poor	Disorganized structure and unclear logic. The language is obscure or overly colloquial and unprofessional.
1 point	Very Poor	The plan is completely incomprehensible, with disorganized and chaotic content.

Supplementary Dimension: TCM Design (Unweighted)

*Core Question*: Does the plan embody the essence of TCM’s syndrome differentiation and treatment, and can it be reasonably integrated with modern rehabilitation medicine?

The evaluation for this dimension is not included in the total score and serves only as an assessment of a distinctive capability. Its detailed scoring rubric is presented in Table 10.

**Table 10:** Detailed Scoring Rubric for Supplementary Dimension: Traditional Chinese Medicine (TCM) Design

Score	Level	Specific scoring criteria
5 points	Excellent	*Profoundly embodies the essence of “Bian Zheng Lun Zhi” (syndrome differentiation and treatment).* Can accurately identify the core TCM syndrome based on the detailed clinical presentation and make personalized modifications. The logic connecting theory, method, formula, and herbs (or acupoints/techniques) is rigorous and interconnected. *Proactively explains the synergistic effects* and precautions of integrating TCM and Western medicine and incorporates holistic concepts such as emotional regulation and dietary guidance.
4 points	Good	*Can accurately perform syndrome differentiation* and formulate a treatment principle and prescription accordingly. The plan clearly classifies the patient’s condition into one or two primary syndromes and provides a corresponding, classic treatment plan (e.g., standard formulas, common acupoint combinations). *Integrates the TCM component with the modern rehabilitation component well,* without obvious conflicts.
3 points	Fair	*Demonstrates an awareness of syndrome differentiation, but it is rather general.* Can propose a fundamentally reasonable TCM syndrome, but the plan tends towards a “disease-specific formula” or an “empirical formula” based on the disease name, lacking sufficient individualization. The content of the plan is generally safe but lacks deep integration.
2 points	Poor	*Lacks a syndrome differentiation process; it is merely a collection of therapies.* The plan simply lists some potentially effective TCM therapies (e.g., “can be supplemented with acupuncture,” “Chinese herbal medicine is recommended”) without providing a basis in syndrome differentiation or a specific plan, or the proposed plan has a weak correlation with the patient’s condition.
1 point	Very Poor	*Conceptual errors or potential risks exist.* The plan shows a misunderstanding of TCM concepts, recommends a TCM therapy that is clearly contraindicated for the patient’s condition (e.g., using strong blood-activating methods for a patient with bleeding tendencies), or the content is entirely a collection of irrelevant, empty jargon. *Alternatively, the plan contains no TCM design.*

## Appendix C

### Online evaluation platform demonstration

The main interface of the online evaluation platform is shown in Fig. 5.

## Abbreviations

LLMs: Large language models
AI: Artificial Intelligence
LMM: Linear Mixed-Effects Models
ICF: International Classification of Functioning, Disabilityand Health
JAMA: Journal of the American Medical Association
DECIDE-AI: Developmental and Exploratory Clinical Investigationsof Decision support systems driven by ArtificialIntelligence
SRQR: Standards for reporting qualitative research
MRI: Magnetic resonance imaging
TCM: Traditional Chinese Medicine
C&S: Clinical Applicability and Safety
S&E: Scientific Rigor and Evidence-Based Conformity
I&C: Individualization and Clinical Problem-Solving
C&L: Clarity and Logicality
ICC: Intraclass Correlation Coefficient
CI: Confidence Interval
SSP: Significantly Superior Performer
ICU: Intensive Care Unit
NPO: Nil Per Os (Nothing by Mouth)
SIMV: Synchronized Intermittent Mandatory Ventilation
rTMS: Repetitive transcranial magnetic stimulation
MBI: Modified Barthel Index
